# Antimalarial and antioxidant activities of novel artesunate-ellagic acid hybrid compound *in vitro* and *in vivo*

**DOI:** 10.3389/fphar.2024.1192659

**Published:** 2024-06-18

**Authors:** Ahmed A. Ishola, Joseph O. Adebayo, Isabela P. Ceravolo, Habibu Tijjani, Edson S. Bento, Henrique F. Goulart, Alessandre C. Crispim, Elizabeth A. Balogun, Antonio E. G. Santana, Antoniana U. Krettli

**Affiliations:** ^1^ Department of Biochemistry, University of Ilorin, Ilorin, Nigeria; ^2^ Malária Experimentale Humana, Instituto René Rachou, Fundacao Oswaldo Cruz, Belo Horizonte, Mato Grosso, Brazil; ^3^ Department of Biochemistry, Bauchi State University, Gadau, Nigeria; ^4^ Instituto de Quimica e Biotecnologia, Universidade Federal de Alagoas (UFAL), Maceio, Alagoas, Brazil; ^5^ Laboratório de Pesquisa Em Recursos Naturais (LPqRN), Campus de Engenharias Ciencias Agrárias, Rio Largo, Brazil

**Keywords:** antioxidant, ellagic acid, artesunate, antimalarial, hybrid compound

## Abstract

**Introduction:** Emergence of drug resistant strains of *Plasmodium* species has necessitated the search for novel antimalarials with unique mechanisms of action. Synthesis of hybrid compounds has been one approach to tackling this challenge. In this study, the synthesis of artesunate-ellagic acid hybrid compound (EA31) from ellagic acid and artesunate and its evaluation for antimalarial and antioxidant activities using *in vitro* and *in vivo* models were carried out.

**Method:** EA31 was synthesized from artesunate and ellagic acid. The activities of the hybrid compound against Plasmodium falciparum W2 and P. berghei NK65 were evaluated, and its antioxidant activities were also determined.

**Results:** The results revealed that EA31 was more active against *P. falciparum* W2 (chloroquine resistant) clone and less cytotoxic to buffalo green monkey kidney cell line compared to artesunate. EA31 was also active against *Plasmodium berghei* NK65 *in vivo*. The results revealed inhibition of β-hematin formation as one of the mechanisms of action of EA31. EA31 also exhibited antioxidant activities.

**Conclusion:** The results revealed that EA31 may exert dual action of killing malaria parasite and mopping the reactive oxygen species that mediate the secondary complications of malaria.

## Introduction

Malaria remains the deadliest human parasitic disease with an estimated 247 million cases worldwide leading to an estimated 619,000 deaths (World Health Organization, 2019). Africa remains the worst-hit region accounting for 95% of the reported cases and 94% of total death recorded globally (World Health Organization, 2019). Children under the age of 5 years remained the most vulnerable globally, accounting for 76.8% of malaria deaths (World Health Organization, 2019). Malaria is caused by the protozoan of the family Plasmodium borne by Anopheles mosquitoes. Of the five species infecting humans, *Plasmodium falciparum* remains the most lethal, accounting for more of the morbidity and mortality in endemic areas (Tibon et al., 2020; Wadi et al., 2020). Current approaches to the prevention and treatment of malaria include: (i) use of antimalarials for prophylaxis, therapy and transmission blockage; and (ii) vector control strategies, including the use of insecticide-treated bednet, indoor residual spraying, etc (Wadi et al., 2020). However, progress on current initiatives have been hampered due to resistance of mosquitoes to insecticides (Cook et al., 2018; Murray et al., 2020) and the emergence of resistant strains of *Plasmodium* species (Mok et al., 2011).

Presently, antimalarial combination therapies, involving two or more drugs with different mechanisms of action, are administered simultaneously to give a synergistic effect against the parasite to reduce the chances for resistance development. The WHO-recommended combination therapies are the artemisinin-based combination therapies (ACTs), in which one of the artemisinin derivatives is used in combination with a partner drug such as amodiaquine, piperaquine, mefloquine and lumefantrine (World Health Organization, 2015). However, the emergence of artemisinin resistance, first reported in Cambodia (Noedl et al., 2008) and later in Southeast Asia (Hien et al., 2012), threatens to become a main problem in the quest for malaria eradication. Artemisinin resistance is evinced as increased survival and slowed clearance of young ring-stage parasites after intense exposure to artemisinin or its derivatives (Marapana and Cowman, 2020). Since then, scientists have been working round the clock towards developing appropriate combinations that would prevent the development and spread of resistance.

New antimalarial drug combinations currently under investigation are developed based on three main principles, which include: (1) optimizing new dose regimens or formulations of some agents; (2) combination therapies, including new agents such as artesunate-pyronaridine, dihydroartemisinin-piperaquine, artemisinin-naphthoquine and arterolane-piperaquine; and (3) new combinations of older agents such as artesunate-mefloquine, artesunate-atovaquone-proguanil and artesunate-chlorproguanil-dapsone (Mishra et al., 2017). However, covalent bitherapy, involving the linking of two distinct pharmacophores which act on different/similar biological targets through different mechanisms of action, is a new approach mainly employed to produce novel hybrid molecules with amplified functions (Meunier, 2008; Muregi and Ishih, 2010; Agarwal et al., 2017). Such a rational approach is also considered an improvement to the current combination therapies whereby two or more drugs are co-formulated into a single dosage form (Tibon et al., 2020). Reports so far indicate that hybrid molecules are effective against all *Plasmodium* species including resistant strains (Lödige and Hiersch, 2015). Notably, MEFAS, a hybrid compound of mefloquine and artesunate, was highly effective against chloroquine-resistant strains and also exhibited low cytotoxicity (Varotti et al., 2008; Penna-Coutinho et al., 2016). Peptide-quinine hybrid compounds have also been synthesized. The most active against *P. falciparum* 3D7 was hybrid Z-L-asp (Bn)-Quinine (IC_50_: 17 nM) comparing favourably well with quinine (IC_50_: 18 nM) (Panda et al., 2013). Dihydroartemisinin-carboxylic acid derivative of quinine hybrid compound was synthesized and found to be more active against *P. falciparum* 3D7 and FcB1 than artemisinin, quinine and 1:1 combination of artemisinin and quinine (Walsh et al., 2007). A primaquine-artemisinin hybrid compound was also synthesized and found to be as active against *P*. *falciparum* W2 (IC_50_: 9 nM) as artemisinin (8 nM) and more active than primaquine (3.3 µM) (Capela et al., 2011). Also, amodiaquine-ellagic acid hybrid was reported to possess better antiplasmodial activity than ellagic acid alone (Żesławska et al., 2014).

Ellagic acid, a gallic acid dimer, is a polyphenol (Debnath et al., 2020) found in its free form, in a series of ellagitannins or as glucoside in plants, especially in fruits and nuts (Jakobek et al., 2009). This molecule has been isolated from several plants, including *Quercus alba*, *Quercus robur* (Mattila et al., 2000), and the West African Tall Variety of *Cocos nucifera* (Silva et al., 2013). Earlier studies have reported the radical scavenging (Priyadarsini et al., 2002), antioxidant (Marković et al., 2013), anti-inflammatory and anticancer activities (Baradaran Rahimi et al., 2020) of the compound. Also, its antiplasmodial activity and potentiation of antimalarials, such as chloroquine, mefloquine and artesunate, have been reported (Soh et al., 2009). However, the use of ellagic acid as a single antimalarial has been hampered due to its low bioavailability (Soh et al., 2009). Thus, the ability of ellagic acid to potentiate the activities of antimalarials, such as artesunate, a drug clinically used for the treatment of malaria due to its rapid parasite clearance activity (Soh et al., 2009), *in vivo* would be limited. However, the formation of hybrid compounds from ellagic acid and antimalarials may circumvent this problem. Thus, this study was carried out to synthesize a single hybrid compound from artesunate and ellagic acid and evaluate it for antimalarial and antioxidant activities *in vitro* and *in vivo*.

## Materials and methods

Ellagic acid and artesunate were purchased from Zelang Medical Technology, China. Artesunate chloride was obtained from Tuyil Pharmaceutical, Ilorin, Nigeria. Sodium carbonate (Na_2_CO_3_), Giemsa stain, chloroquine diphosphate, 2, 2-diphenyl-1-picrylhydrazyl, hemin, sodium acetate, acetic acid, methanol (MeOH-d_4_) and dimethyl sulfoxide (DMSO-d_6_) were obtained from Sigma-Aldrich (St Louis, M. EUA). Aluminium sheets pre-coated with silica gel (G60, 0.25), sodium nitroprusside, sulphanilamide, naphthyl ethylenediamine dihydrochloride, potassium ferricyanide, ammonium molybdate, sodium citrate, dichloromethane, methanol, immersion oil, sulphosalicylic acid, sodium azide, formaldehyde, butylated hydroxytoluene (BHT) were obtained from Merck^®^ (Merck Darmstadt, Germany). Other reagents/chemicals were of analytical grade and were prepared according to specifications.

### Parasite strain and experimental animals

Chloroquine-sensitive strain of *Plasmodium berghei* NK65 was acquired from the Institute for Advanced Medical Research and Training, University College Hospital, Ibadan, Nigeria. The parasites were maintained in mice by serial passages of blood from the infected donor mouse to the naive recipient. Adult Swiss albino mice of average weight of 20.45 ± 2.01 g were acquired from the Animal Holding Unit of the Department of Biochemistry, Faculty of Life Sciences, University of Ilorin, Ilorin, Nigeria.

### Ethical clearance

Ethical clearance for the study was obtained from the University of Ilorin Ethical Review Committee (UERC), with the UERC Approval number: UERC/ASN/2015/055.

### Synthesis of hybrid molecules from artesunate and ellagic acid

**SCHEME 1:**
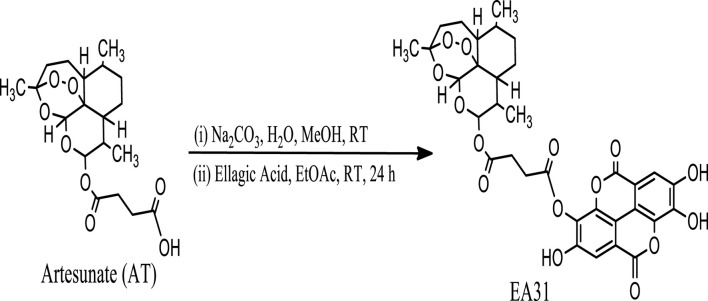
Reaction scheme for the synthesis of the artesunate-ellagic acid hybrid molecule (EA31).

Hybrid molecule (EA31, Scheme 1) was synthesized using a slightly modified method of Varotti et al. (2008). Artesunate (2.00 g, 5.2 mmol) was dissolved in 30 mL H_2_O/MeOH (8:2). With stirring, 30 mL of ethyl acetate was added to this solution at room temperature. Sodium bicarbonate was added until the effervescence ceased. The two phases were separated; the organic layer was dried over anhydrous sodium sulfate, and the solvent evaporated. The free base (0.69 g) obtained was dissolved in 50 mL ethyl acetate, and a solution of ellagic acid (0.70 g, 2.3 mmol) in ethyl acetate was added. The mixture was stirred at room temperature (22°C ± 2°C) for 24 h. The solvent was evaporated and purified on a silica gel column eluted with 100% methanol to give 1.20 g of pure brown solid precipitate. HPLC profiles of reactants and products were carried out using gradient elution with methanol and 0.2% (v/v) phosphoric acid on an automated injector C_18_ HPLC with a UV detector (Shimazu, Kyoto, Japan). Artesunate, ellagic acid and EA31 were monitored using a 254 nm channel. Infrared (IR) spectra were recorded on an IRPrestige21 (Shimadzu Scientific, Kyoto, Japan). Then, 10 mg of sample was dissolved in 0.5 mL of DMSO-d_6_ for various NMR analyses while 1H and 13C-NMR spectra were recorded at room temperature (22°C ± 2°C) on a Bruker^®^ 400 MHz spectrometer (Bruker, Germany) except otherwise stated (Supplementary Figure S1–S6).

### *In vitro* antiplasmodial studies

The candle jar technique of Trager and Jensen (1976) was employed in culturing *P. falciparum* parasites in human erythrocyte (RBC). Briefly, parasites were cultured in Petri dishes (Corning, Santa Clara, CA, United States) containing RPMI 1640 culture medium [supplemented with 1% (v/v) albumax II (Gibco, United States)] with 5% hematocrit. Plates were incubated at 37°C, using the candle jar method. The culture medium was changed daily. The parasite(s) were synchronized using sorbitol as reported by Lambros and Vanderberg (1979) to obtain mainly ring forms, diluted and incubated in a 96 well plate containing the hybrid compound and the standard drug, or culture medium with 0.5% DMSO, used as a positive (+ve) control of parasite growth. The SYBR test was employed as outlined (Smilkstein et al., 2004). Briefly, serial dilutions of EA31 were incubated at 37°C with the parasite suspensions (0.5% parasitemia and 2% hematocrit) in “U” bottom 96-wells plates. After 48 h, the culture supernatant was removed and replaced by 100 µL of lysis buffer solution [Tris (20 mM; pH 7.5), EDTA (5 mM), saponin (0.008%; wt/vol), and Triton X-100 (0.08%; vol/vol)] followed by addition of 0.2 μL/mL Sybr Safe (Sigma-Aldrich, Carlsbad, CA, United States). The plate contents were then pipetted into a flat bottom plate and incubated in the dark for 30 min. The reading was in a fluorimeter (Synergy H4 Hybrid Reader, BioteK) with excitation at 485 nm and emission at 535 nm. Compounds exhibiting IC_50_ values ≤ 10 μg/mL were considered to be active, those exhibiting IC_50_ values in the range of 10 to ≤25 μg/mL were considered moderately active, while those exhibiting IC50 values > 25 μg/mL were considered inactive (Lima et al., 2015).

### Cytotoxicity assay

Cytotoxicity test was carried out with buffalo green monkey (BGM) kidney cell line as reported by Aguiar et al. (2012). Cells were cultured in 75 cm^2^ plates with RPMI 1640 medium supplemented with 10% fetal bovine serum (FBS) and gentamicin 40 mg/mL, at 5% CO_2_ atmosphere and 37°C. The cells were trypsinized when the monolayer was confluent, washed with culture medium, distributed in a flat-bottomed 96-well plate (1 × 10^5^ cells/mL), and incubated at 37°C for 18 h to ensure cell adherence. The cells were properly diluted and incubated with 20 μL of the hybrid molecule (EA31), ellagic acid and artesunate at different concentrations (1–1,000 μg/mL) for 24 h in a 5% CO_2_ atmosphere at 37°C. The neutral red assay as outlined by Borenfreund and Borrero (1984) was used to evaluate cell viability by the accumulation of the dye in the lysosomes of viable cells. Neutral red solution (NRS) (4 mg/mL, 200 μL) was then added to the plates and incubated for 3 h. The supernatant was carefully removed, followed by the addition of 200 μL of formaldehyde (0.5% v/v) and CaCl_2_ (1%) solution. After 5 min, the supernatant was removed and 100 μL of alcohol/acetic acid solution was added to extract the dye. The absorbance was read at a wavelength of 540 nm on an ELISA reader (SpectraMax 340PC384, Molecular Devices). Cell viability was reported as the percentage of control absorbance obtained in untreated cells and MLD_50_ (median lethal dose) was determined. Based on the values of cytotoxicity (MLD_50_) and antimalarial activity (IC_50_), the selectivity index (SI) of activity was calculated using the formula:

SI=MLD50/IC50

### β-hematin inhibition assay

β-Hematin inhibition assay was carried out using the method described by Egan and Ncokazi (2005). Briefly, hematin stock solution was dispensed in a series of Eppendorf tubes (33.6 nmol/Eppendorf tube). Each tube contained 2.02 µL of EA31 solution (prepared by dissolving the drug in 1.0 M HCl). Concentrations were pre-set to give 0–10 equivalents relative to hematin in the final solution. After mixing, 11.74 µL of 12.9 M acetate solution (pH 5.0) pre-incubated for 30 min at 60°C was added. The final hematin concentration was 1 mM and the final pH of the solution was 4.5. Reaction mixtures were incubated at 60°C for 60 min. They were then stopped at room temperature by adding 900 µL of 5% (v/v) pyridine to buffer the mixtures to a final pH of 7.4. This was followed by an addition of 1,100 µL of 5% (v/v) pyridine solution. Solutions were shaken to ensure complete dissolution of hematin and the β-hematin was allowed to settle at ambient temperature for at least 15 min. The precipitate from the walls of the Eppendorf tube was scraped to ensure complete dissolution of hematin. Supernatants were carefully transferred to a cuvette without disturbing the precipitate. Absorbance values were read at 405 nm. The sigmoidal dose-response curve was analyzed by nonlinear least-squares fitting using GraphPad Prism to determine the number of equivalents of drug required to inhibit β-hematin formation by 50% (IC_50_).

### *In vitro* radical scavenging assay

#### 1,1-Diphenyl-2-picrylhydrazyl (DPPH) radical scavenging activity

The free radical scavenging capacity of EA31 was determined by using the method of Hao and Zhaobao (2010). Briefly, various concentrations (1 mL) of the hybrid molecule in methanol were added to 4 mL of 0.1 mmol/L methanolic solution of DPPH. A blank probe was obtained by mixing 4 mL of 0.1 mmol/L methanolic solution of DPPH and 200 μL of deionized distilled water (ddH2O). After 30 min of incubation in the dark at 25°C, the absorbance was read at 517 nm against prepared blank. Butylated hydroxytoluene (BHT) was used as reference.

#### 2,2′-azino-bis(3-ethylbenzothiazoline-6-sulfonic) acid (ABTS) scavenging activity

The ABTS scavenging activity of the EA31 was measured according to the procedure outlined by Re et al. (1999). Briefly, the ABTS^•+^ solution was prepared by a reaction of 7 mM ABTS in H_2_O and 140 mM potassium persulphate, stored in the dark at room temperature (22°C ± 2°C) for 30 min. ABTS^•+^ solution (1 mL) was added to 3 mL of the compound at various concentrations (10–50 μg/mL). After 30 min, the absorbance was read at 734 nm.

#### Nitric oxide (NO) radical scavenging activity

NO scavenging activity was measured using the method of Fiorentino et al. (2008). The assay mixture contained 2 mL of 5 mM sodium nitroprusside (SNP) (in 0.1 M phosphate pH 7.4) and 0.5 mL of ascorbic acid. The assay mixture was incubated at 37°C for 2 h. Thereafter, 0.1 mL of the assay mixture was withdrawn and added to a 96-well microplate, followed by the addition of 0.1 mL of Griess reagent (1% sulphanilamide (C_6_H_8_N_2_O_2_S), 0.1% naphthyl ethylenediamine dihydrochloride in 5% phosphoric acid). The mixture was kept in the dark for 10 min at 25°C, followed by measurement of absorbance at 530 nm. The NO scavenging activity was expressed as % relative to the absorbance of the blank at 530 nm.

#### Ferric-reducing antioxidant assay (FRAP)

The FRAP of EA31 was determined according to the method reported by Girgih et al. (2013). To 250 µL of test compounds or BHT was added 250 µL of 0.2 M phosphate buffer (pH 6.6) and 250 µL of 1% potassium ferricyanide (C_6_N_6_FeK_3_) solution. The mixture was incubated at 50°C for 20 min. Thereafter, 250 µL of 10% aqueous trichloroacetic acid (TCA) was added. Then, to 250 µL of the drug/TCA mixture was added 50 µL of 1.0% FeCl_3_ and 200 µL distilled water. They were allowed to stand at room temperature for 10 min. The mixture was thereafter centrifuged for 20 min at 1,000 x g. From these, 200 µL of clear supernatant was transferred to a clear bottom 96-well plate, and absorbance was measured at 700 nm.

#### Total antioxidant capacity

The total antioxidant capacity of EA31 was evaluated using the phosphomolybdenum assay (Prieto et al., 1999). Briefly, the test compound (0.1 mL) was combined with 1 mL reagent solution [0.6 M Tetraoxosulphate (VI) acid (H_2_SO_4_), 28 mM sodium phosphate and 4 mM ammonium molybdate (NH4)_6_Mo_7_O_24_]. The mixture was incubated at 95°C for 90 min. After cooling to room temperature (22°C), 200 µL of the mixture was transferred to a clear bottom 96-well plate, and absorbance was measured at 695 nm.

#### Suppressive antimalarial test

The 4-day suppressive test (Peters, 1965) with some modifications (Carvalho et al., 1991) was employed for the determination of the antimalarial activity of the synthesized hybrid compound (EA31). Forty-five adult outbred Swiss mice were inoculated intraperitoneally with *P. berghei* NK65, a chloroquine-sensitive strain. Tail blood was obtained from a donor mouse of known parasitemia into a sample bottle containing 2 mL 3.8% citrate/5% glucose solution. This was then diluted appropriately to obtain an inoculum size of 1 × 10^5^ infected red blood cells in 200 µL which was used to inoculate each mouse. The infected animals were randomly divided into nine groups of five per cage and daily treated with 200 µL of the various doses of the drugs by the oral route for three consecutive days. Group A mice (control) were administered 5% DMSO, mice of groups B, C, D, E, F were administered 5, 10, 20, 40 and 80 mg/kg body weight of EA31 respectively while mice of groups G, H and I were administered 10 mg/kg body weight of ellagic acid, 4 mg/kg body weight of artesunate and 20 mg/kg body weight of chloroquine respectively. Overall mortality was monitored daily until day 30 post-inoculation. Parasitemia and inhibition of parasite growth in the hybrid-treated group in relation to the non-treated control group were calculated. Compounds with 50% inhibition of parasite growth were considered active, those with 30%–50% inhibition of parasite growth were considered partially active while those with <30% inhibition of parasite growth were considered inactive. Also, effective doses (ED_50_ and ED_90_) of hybrid compound were determined.

#### Curative antimalarial test

Curative (Rane) test was performed as reported by Ryley and Peters (1970). Forty-five mice were infected intraperitoneally on Day 0 and left for 72 h before the commencement of treatment. The mice were randomly divided into nine groups of five mice each. Groups A, B, C, and D were orally administered 5% DMSO, 20 mg/kg body weight of chloroquine, 4 mg/kg body weight of artesunate and 10 mg/kg ellagic acid respectively while groups E, F, G, H, and I were orally administered 5, 10, 20, 40, and 80 mg/kg body weight of the hybrid compound (EA31) respectively for four consecutive days. Parasitemia was calculated from the thin blood smears prepared from the tail blood of mice. Also, the mice were monitored for 30 days for mortality and the mean survival time (MST) was calculated.

#### *In vivo* antioxidant assay

The method described by Ryley and Peters (1970) was adopted in inducing oxidative stress prior to the evaluation of the effects of the hybrid compound (EA31) on the antioxidant system in mice. Sixty-four mice were randomly divided into eight groups of eight mice each. Out of the 64 mice, 56 were infected intraperitoneally with 1 × 10^5^ infected red blood cells in 200 μL inoculum and kept together for 24 h. The infected mice were then randomly divided into seven groups (E–H) of eight mice each and treated daily by the oral route for three consecutive days with different doses of the hybrid molecule, ellagic acid and 5% DMSO. Groups A (uninfected control) and B (infected animals) were administered 200 μL of 5% DMSO solution, group C was administered 4 mg/kg body weight ellagic acid while Groups D, E, F, G, and H were administered 5, 10, 20, 40 and 80 mg/kg body weight of Artesunate-ellagic acid Hybrid. Twenty-four hours after the last administration of the drugs, four animals in each group were sacrificed under slight diethyl ether anesthesia and were then dissected. Venous Blood was collected into clean, dry, sterile sample tubes containing EDTA and centrifuged at 3,000 rpm for 5 min at 4°C to remove plasma. The supernatant and the buffy coat were aspirated while red blood cells were washed with phosphate buffer (0.1 M, pH 7.4). The red blood cells were then lysed using the repeated freeze-thaw procedure and the lysate was stored at −20°C. The liver was homogenized in ice-cold 0.25 M sucrose solution (1:5, w/v) and the homogenate was centrifuged at 10,000 rpm for 4 min in a centrifuge (Eppendorf Centrifuge, Model 5,702, Germany). The supernatant was aspirated into new sample bottles and stored overnight at −20°C to ensure maximum release of the enzymes (Ngaha et al., 1989). On day 11 post-inoculation, the remaining mice were also sacrificed and treated similarly. The extent of lipid peroxidation in tissues was assessed by quantifying malondialdehyde (MDA) concentration as described by Varshney and Kale (1990). The method reported by Misra and Fridovich (1972) was used to determine superoxide dismutase (SOD) activities in tissues. The method of Sinha (1972) was used to determine catalase (CAT) activity. The activities of Glutathione peroxidase (GPx) in tissues were evaluated by the procedure of Rotruck et al. (1973). The method described by Goldberg and Spooner (1983) was used to determine glutathione reductase (GR) activity. The levels of reduced glutathione (GSH) in the tissues were estimated by the method of Beutler et al. (1963).

#### Statistical data analysis

Data were expressed as mean values ±SEM of five replicates except otherwise stated. All results were statistically analyzed using one-way ANOVA, followed by Duncan Multiple Range Test. Differences between group means were considered significant at *p* < 0.05. Graphs were generated with GraphPad Prism six software (GraphPad Software, California, United States) and Origin v7.022 Software (OriginLab Corporation, Northampton, United States).

## Results

### Structure of artesunate-ellagic acid hybrid (EA31)

**FIGURE 1:**
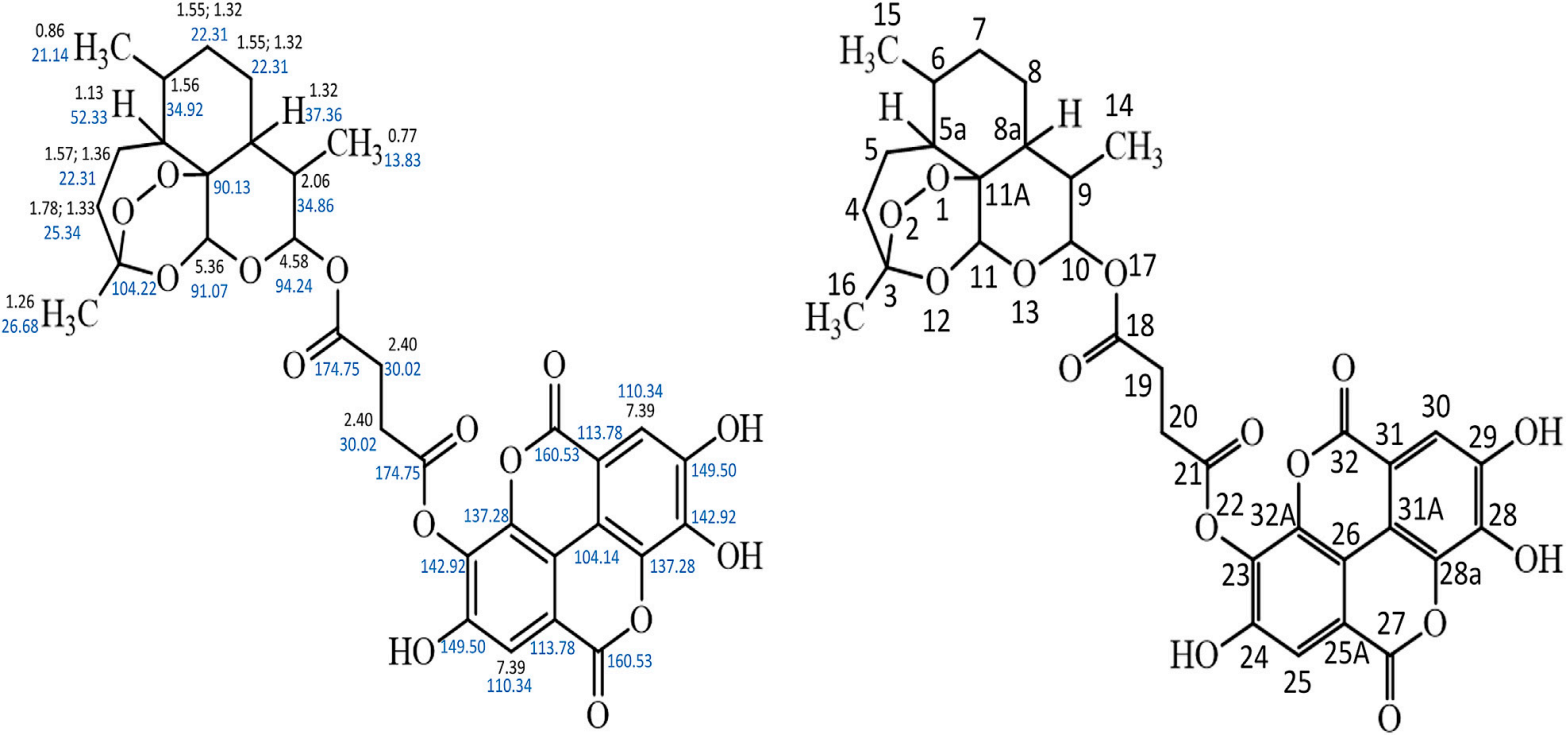
Artesunate-Ellagic acid hybrid compound.

The HPLC spectrum revealed a single peak confirming the presence of a compound and not a blend of two compounds. The ^13^C NMR spectrum showed the presence of 32 different carbon environment while the 1H NMR spectrum revealed the presence of 17 protons in the hybrid molecule. The carbon atoms were distinguished based on the electron shielding effect, with C-21 and C-18 exhibiting the greatest chemical shift on the spectra. The infra-red spectrum revealed various functional groups in the hybrid molecule. These include C = O, C–H, C = H and–OH. The COSY spectrum revealed the proton-proton relationship, while the HMBC spectrum revealed the carbon-proton relationship. The structure of the artesunate-ellagic acid hybrid compound (Figure 1) was elucidated from the various spectra and the combined NMR data (Supplementary Figures S1–S6) as shown below:

Artesunate-Ellagic acid hybrid (EA31): Chemical formula C_33_H_32_O_15_; IR (υ_max/cm_): 3,373, 2,922, 1,697, 1,618, 1,018; R_f_: 0.23; R_t_: 34.33 min ^1^H NMR (DMSO-d_6_), δ 0.77 (d, *J* = 7.20 Hz, 3H), 0.86 (d, *J* = 6.34 Hz, 3H), 1.13 (m, 1H), 1.26 (s, 3H), 1.33–1.78 (m, 2H), 1.36–1.57 (m, 2H), 1.56 (m, 1H), 1.32 (m, 1H) 1.32–1.55 (m, 4H), 2.06, 2.40 (s, 4H), (m, 1H), 4.58 (d, *J* = 9.09, 1H), 5.36 (s, 1H), 7.39 (s, 2H), ^13^C NMR (DMSO-d_6_), δ 104.22 (O-C-CH_2_-), 25.34 (−CH_2_-), 22.31 (−CH_2_−) (3x), 52.33 (−CH-), 34.92 (−CH-CH_3_), 37.36 (−CH-), 34.86 (−CH-), 94.24 (−O-CH-O-), 90.13 (−C-), 91.07 (−O-CH-O), 13.83 (-CH_3_), 21.14 (−CH_3_), 26.68 (−CH_3_), 174.75 (−C=O) (2x), 30.02 (−CH_2_−) (2x), 142.92 (−O-C = C-) (2x), 149.50 (HO-C = C-) (2x), 110.40 (−C-C = C-) (2x), 113.78 (−C-C=C) (2x), 104.14 (−C-C=C) (2x), 160.53 (−C-CO-C-) (2x), 137.28 (−C = C-C-) (2x) (Supplementary Table S1; Figure 1).

### *In vitro* radical scavenging activity

**FIGURE 2:**
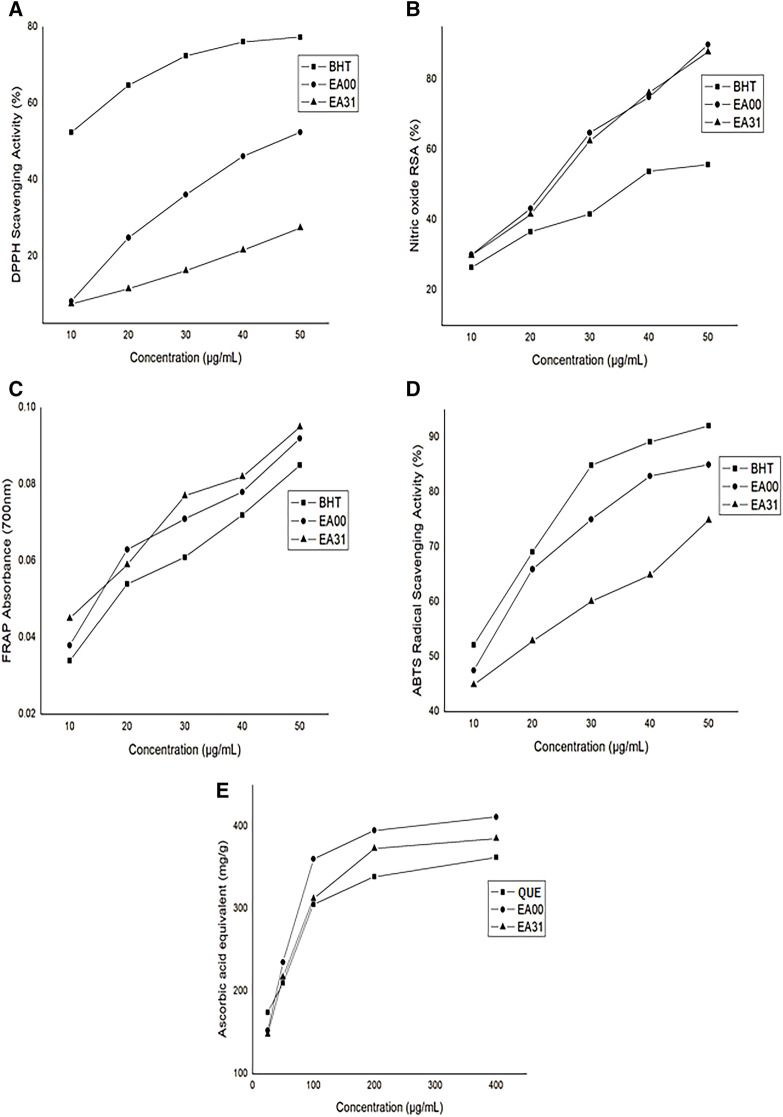
*In vitro* antioxidant activity of ellagic acid-artesunate hybrid compound **(A)** DPPH radical scavenging activity **(B)** Nitric oxide radical scavenging activity **(C)** Ferric reducing antioxidant power **(D)** ABTS radical scavenging activity **(E)** Total antioxidant capacity. Values are Means ± SEM of four determinations. BHT, Butylated hydroxytoluene; EA00, Ellagic acid; EA31, Ellagic acid-Artesunate Hybrid Molecule. QUE, Quercetin; ABTS, 2,2′-azino-bis (3-ethylbenzothiazoline-6-sulfonic) acid; DPPH, 1,1-Diphenyl-2-picrylhydrazyl.

The hybrid compound (EA31) had a significantly higher IC_50_ (>50 μg/mL) for DPPH radical scavenging activity compared to BHT (<10 μg/mL) and the parent compound ellagic acid (47 μg/mL) (Figure 2A). Nitric oxide (NO) scavenging activity of the hybrid compound was higher, having lower IC_50_ (24.75 μg/mL) compared to BHT (38 μg/mL) but lower compared to ellagic acid (23.75 μg/mL) (Figure 2B). EA31 exhibited a higher ferric-reducing antioxidant power than BHT (Figure 2C). However, the ABTS radical scavenging activity of EA31 was lower (IC_50_ = 17.5 μg/mL) than that of ellagic acid (11.5 μg/mL) and BHT (<10 μg/mL) (Figure 2D). The total antioxidant capacity of EA31 was higher than that of quercetin but lower than that of ellagic acid, (Figure 2E).

### Antiplasmodial activity and cytotoxicity

EA31 elicited lower IC_50_ against the *P. falciparum W2 clone* (Table 1) compared to artesunate and ellagic acid, exhibiting approximately two-fold the activity of artesunate and one hundred and sixty-two-fold the activity of ellagic acid. Cytotoxicity studies on EA31 showed that the hybrid compound had a higher MLD_50_ (301 μg/mL**)** compared to artesunate and ellagic acid (115 and 33 μg/mL, respectively) (Table 1). Consequently, the highest selectivity index was obtained for EA31 (41,805.56), which was seventeen-fold that of artesunate and four hundred and twenty-six-fold that of ellagic acid (Table 1). The results also revealed that EA31 exhibited β-hematin inhibitory activity, comparing favourably well with chloroquine (IC_50_ of 5.86 μg/mL and 5.67 μg/mL, respectively; Table 2).

**TABLE 1:** Antiplasmodial activity and cytotoxicity of artesunate-ellagic acid hybrid molecule (EA31) and parent compounds.

Compounds (µg/mL)	Cytotoxicity against BGM cell line MLD_50_ (µg/mL) (X ± SD)	Activity against *P. falciparum* W_2_ strain IC_50_ (µg/mL) (X ± SD)	Selectivity Index**	Active
Elagic acid	115 ± 19	1.1666 ± 0.058	98.58	Yes
Artesunate	33 ± 9	0.0134 ± 0.006	2462.69	Yes
EA31	301 ± 36	0.0072 ± 0.003	41805.56	Yes

Values are means ± SD of results of 3 experiments

** Selectivity index (SI) = MLD_50_/IC_50_. Drugs with SI <10 are considered toxic.

BGM cell line: Blue Green Monkey kidney cell line

**TABLE 2:** Inhibitory activity of artesunate-ellagic acid hybrid molecule (EA31) against β-hematin formation.

Compounds	% inhibition					IC_50_ (µg/mL)
	2 μg/mL	4 μg/mL	6 μg/mL	8 μg/mL	10 μg/mL	
CQ	23.19 ± 1.75^a^	47.84 ± 4.13^b^	71.26 ± 2.05^c^	85.31 ± 7.12^d^	90.24 ± 3.23^e^	5.67
EA31	21.26 ± 2.46^a^	48.51 ± 3.47^b^	69.37 ± 3.73^c^	83.25 ± 5.35^d^	91.49 ± 2.19^e^	5.86

Values are means ± SEM of three replicates. Values with different alphabet superscripts in each column are significantly different (*p* < 0.05).

### Antimalarial activities

Suppressive antimalarial study revealed a higher chemosuppression for EA31 compared to its parent compounds (i.e., ellagic acid and artesunate) at doses higher than 5 mg/kg body weight and compared favorably well with chloroquine at doses higher than 10 mg/kg body weight on day 4 post-inoculation (Table 3). At the dose of 20 mg/kg body weight, EA31 and chloroquine exhibited chemosuppression of 90.72% and 93.81% respectively on day 4 post-inoculation. EA31 increased the mean survival time of infected mice by 5, 12, 11, 15, and 14 days at the doses of 5, 10, 20, 40 and 80 mg/kg respectively compared to the negative control (Table 3). For curative study, EA31 elicited higher chemosuppression compared to artesunate at doses higher than 20 mg/kg body weight and at all doses compared to ellagic acid (Table 4). The hybrid compound had lower chemosuppression compared to chloroquine on days 7, 9, and 11 post inoculation (Table 4). EA31 increased the mean survival time of infected mice by 5, 7, 7, 6 and 9 days at the doses of 5, 10, 20, 40 and 80 mg/kg body weight respectively compared to negative control (Table 4). For the suppressive study, EA31 elicited the lowest ED_50_ and ED_90_ (8.88 and 15.98 mg/kg body weight) on day 4 post-inoculation (Table 5). Furthermore, for the curative study, the hybrid compound elicited the lowest ED_50_ and ED_90_ (13.26 and 23.89 mg/kg body weight) on day 7 post-inoculation (Table 5).

**TABLE 3:** Parasitemia in *Plasmodium berghei* NK65-infected mice treated with artesunate-ellagic acid hybrid molecule (EA31).

Groups	Parasitaemia (% chemosuppression)			
	Day 4*	Day 6*	Day 8*	MST
Control (Untreated)	0.97	4.03	5.56	14
20 mg/kg b.w. Chloroquine	0.06 (93.81)	0.45 (88.83)	0.52 (90.65)	22
4 mg/kg b.w. Artesunate	0.27 (72.16)	0.89 (77.92)	1.92 (65.47)	19
10 mg/kg b.w. Ellagic Acid	0.35 (63.92)	2.11 (47.64)	3.34 (39.93)	21
5 mg/kg b.w. EA31	0.34 (64.95)	1.78 (55.83)	3.15 (43.34)	19
10 mg/kg b.w. EA31	0.14 (85.56)	1.40 (65.26)	2.09 (62.41)	26
20 mg/kg b.w. EA31	0.09 (90.72)	1.29 (68.00)	2.01 (63.85)	25
40 mg/kg b.w. EA31	0.05 (94.85)	1.12 (72.21)	1.57 (71.76)	29
80 mg/kg b.w. EA31	0.03 (96.91)	0.75 (81.39)	1.43 (74.28)	28

Values are means of 5 replicates. *Day post-inoculation; b.w.: body weight

**TABLE 4:** Activity of artesunate-ellagic acid hybrid molecule (EA31) against established infection of *Plasmodium berghei* NK65 in mice

Groups	Parasitaemia (% chemosuppression)			
	Day 7*	Day 9*	Day 11*	MST
Control (Untreated)	2.10	3.31	4.37	12
20 mg/kg b.w. Chloroquine	0.17 (91.90)	0.26 (92.15)	0.42 (90.39)	28
4 mg/kg b.w. Artesunate	0.54 (74.29)	1.26 (61.93)	2.51 (42.56)	17
10 mg/kg b.w. Ellagic Acid	1.45 (30.95)	2.49 (24.77)	4.01 (8.23)	16
5 mg/kg b.w. EA31	0.81 (61.43)	1.72 (48.04)	3.02 (30.89)	17
10 mg/kg b.w. EA31	0.62 (70.48)	1.49 (54.98)	2.95 (32.49)	19
20 mg/kg b.w. EA31	0.39 (81.43)	1.24 (62.54)	2.38 (45.54)	19
40 mg/kg b.w. EA31	0.33 (84.29)	1.13 (65.86)	1.99 (54.46)	18
80 mg/kg b.w. EA31	0.28 (86.67)	1.02 (69.18)	1.86 (57.43)	21

Values are means of 5 replicates. b.w., body weight; *Day post-inoculation

**TABLE 5:** Effective doses (ED_50_ and ED_90_) of artesunate-ellagic acid hybrid molecule (EA31) in *Plasmodium berghei* NK65-infected Mice.

Effective doses		
Days post-inoculation	ED_50_ (mg/kg b.w.)	ED_90_ (mg/kg b.w)
**Suppressive test**		
Day 4	8.88	15.98
Day 6	22.90	41.22
Day 8	9.06	16.20
**Curative test**		
Day 7	13.26	23.89
Day 9	14.80	26.64
Day 11	18.95	34.11

b.w.; body weight

### *In vivo* antioxidant activities

**FIGURE 3:**
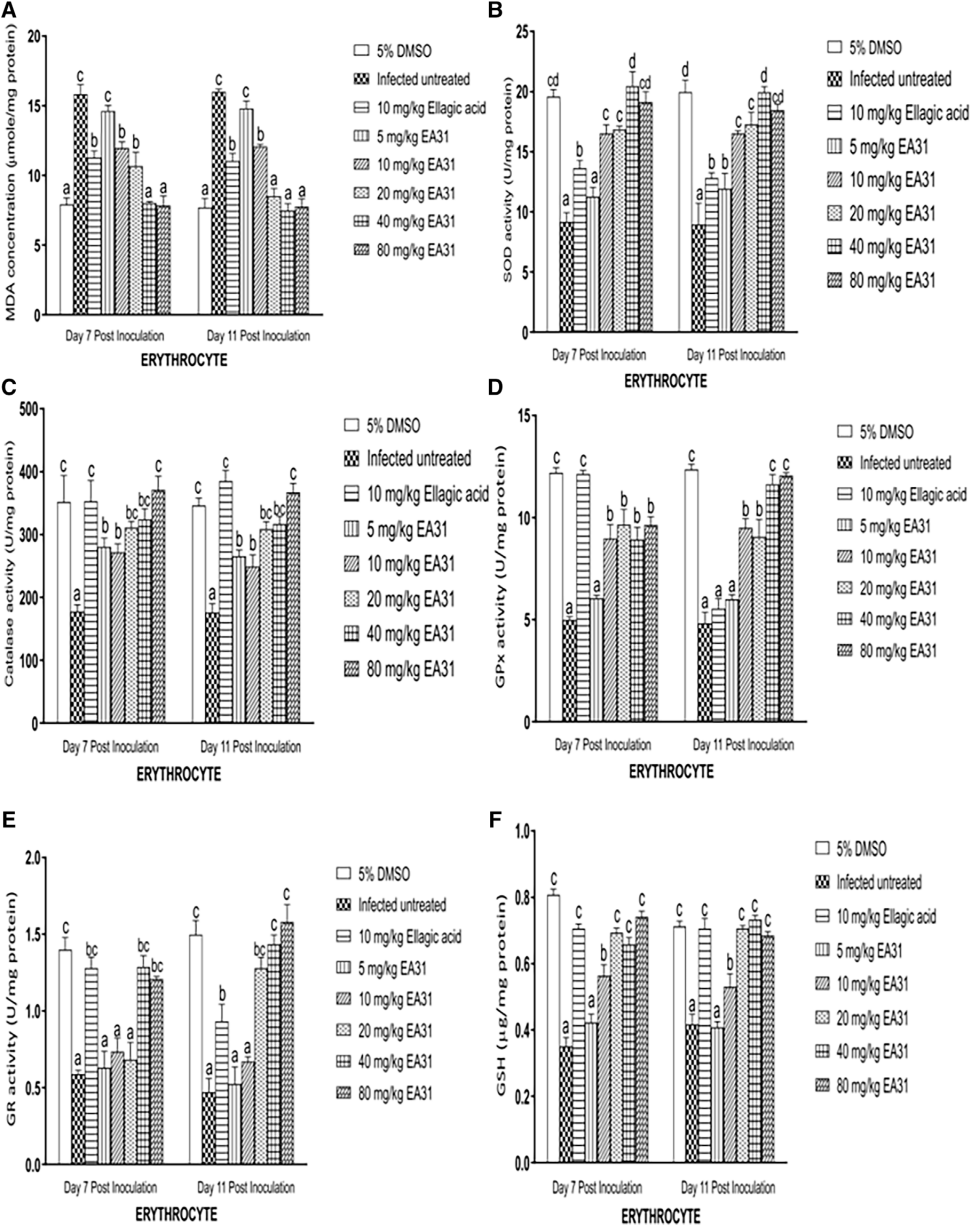
Effect of artesunate-ellagic acid hybrid on *in vivo* antioxidant parameters in the erythrocyte of Plasmodium berghei NK65-infected mice **(A)** malondialdehyde concentration **(B)** superoxide dismutase activity **(C)** catalase activity **(D)** glutathione peroxidase activity **(E)** glutathione reductase activity **(F)** reduced glutathione concentration. Values are means ± SEM of four determinations. Bars for each day with different alphabets are significantly different (*p* < 0.05).

On days 7 and 11 post-inoculation, erythrocyte MDA concentration was significantly increased (*p* < 0.05) in negative control by 100.1% and 107.8% respectively compared to normal control (Figure 3A). However, on day 7 post-inoculation, EA31 significantly reduced (*p* < 0.05) erythrocyte MDA concentration at doses higher than 5 mg/kg body weight compared to negative control, reverting it to the range of normal control at 40 and 80 mg/kg body weight (49.3% and 50.5% reduction respectively). The same trend was observed on day 11 post-inoculation but doses of EA31 higher than 10 mg/kg body weight were able to revert the observed increase in erythrocyte MDA concentration of negative control to the range of normal control. *P. berghei* infection caused a significant decrease (*p* < 0.05) in SOD activity in the erythrocyte of negative control compared to normal control on days 7 and 11 post-inoculation (Figure 3B). Administration of EA31 significantly increased (*p* < 0.05) SOD activity in the erythrocyte by 123.1% and 108.7% at doses of 40 and 80 mg/kg body weight respectively on day 7 post-inoculation and by 92.9%, 122.7%, 106.2% at doses of 20, 40 and 80 mg/kg body weight respectively on day 11 post-inoculation compared to negative controls (Figure 3B). On days 7 and 11 post-inoculation, *P*. *berghei* infection caused significant decrease (*p* < 0.05) in erythrocyte CAT activity in negative control compared to normal control (Figure 3C). Treatment with EA31 significantly reverted (*p* < 0.05) the observed reduction in CAT activity on days 7 and 11 post-inoculation, with 75.5%, 82.3% and 109.3% increase at 20, 40 and 80 mg/kg body weight on day 7 post-inoculation compared to negative control and a similar trend was observed on day 11 post-inoculation (Figure 3C). *P. berghei* NK65 infection caused a significant decline (*p* < 0.05) in erythrocyte GPx activity of negative control compared to normal control on days 7 and 11 post inoculation (Figure 3D). Treatment with EA31 reverted the observed reduction by significantly increasing (*p* < 0.05) erythrocyte GPx activity by 93.6% at 20 mg/kg on day 7 post-inoculation and 87.3% at 20 mg/kg on day 11 post-inoculation compared to negative controls. Inoculation with *P. berghei* NK65 significantly reduced (*p* < 0.05) erythrocyte GR activity by 138.2% on day 7% and 218.8% on day 11 post-inoculation in negative control compared to normal control (Figure 3E). No significant alteration was observed in erythrocyte GR activity at lower doses of EA31 compared to negative controls on days 7 and 11 post-inoculation. Nevertheless, erythrocyte GR activity was significantly increased (*p* < 0.05) by 119% and 105.6% at 40 and 80 mg/kg body weight respectively on day 7 post-inoculation compared to negative control, a trend that continued till day 11 where EA31 caused over 200% increase (*p* < 0.05) in erythrocyte GR activity at doses above 20 mg/kg body weight (Figure 3E). *P. berghei* NK65 infection imposed a significant decline (*p* < 0.05) in erythrocyte GSH concentration by 56% and 42% on days 7 and 11 post-inoculation respectively compared to normal controls (Figure 3F). EA31 significantly reverted (*p* < 0.05) this trend by causing over 75% increase in erythrocyte GSH concentration at doses higher than 10 mg/kg on days 7 and 11 post-inoculation compared to negative controls.

**FIGURE 4:**
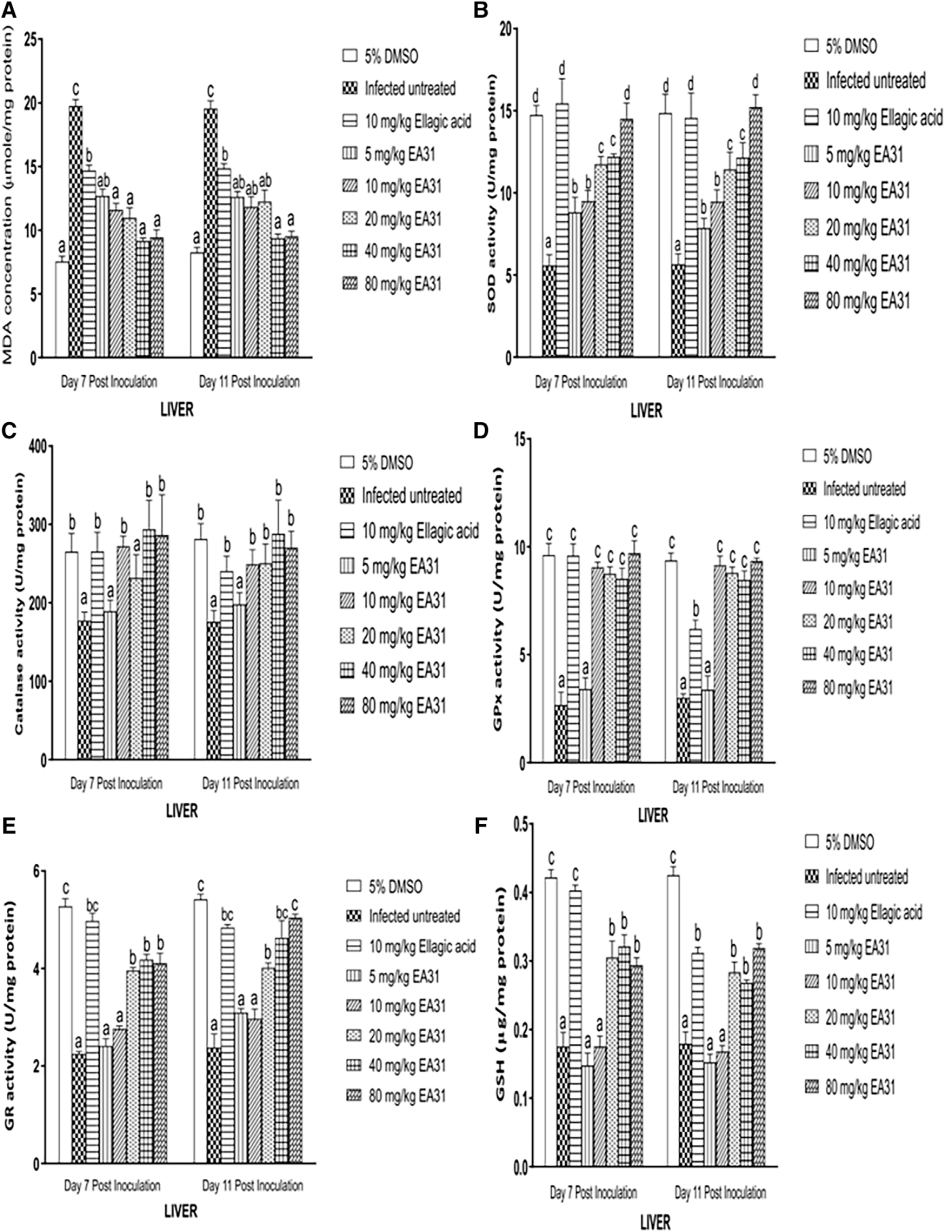
Effect of artesunate-ellagic acid hybrid on *in vivo* antioxidant parameters in the liver of Plasmodium berghei NK65-infected mice **(A)** malondialdehyde concentration **(B)** superoxide dismutase activity **(C)** catalase activity **(D)** glutathione peroxidase activity **(E)** glutathione reductase activity **(F)** reduced glutathione concentration. Values are means ± SEM of four determinations. Bars for each day with different alphabets are significantly different (*p* < 0.05).

Hepatic MDA concentration was significantly increased (*p* < 0.05) by 161.7% and 136.5% in negative control compared to normal control on days 7 and 11 post-inoculation respectively (Figure 4A). Hepatic MDA concentration was significantly reduced (*p* < 0.05) by 53.62% on day 7% and 62.03% on day 11 post-inoculation upon treatment with 40 mg/kg body weight EA31 compared to negative control. *P. berghei* infection caused a significant decrease (*p* < 0.05) in hepatic SOD activity of negative control compared to normal control (Figure 4B). However, EA31 caused significant increase (*p* < 0.05) in hepatic SOD activity by over 100% at doses above 10 mg/kg on days 7 and 11 post-inoculation compared to negative control (Figure 4B). *P. berghei* infection caused a significant decrease (*p* < 0.05) by 31% in hepatic CAT activity in negative control on day 7 post-inoculation, with a further decrease on day 11 post-inoculation, compared to normal control. However, hepatic CAT activity was increased by over 60% on days 7 and 11 post-inoculation upon treatment with dose of 40 mg/kg body weight of EA31 compared to negative control (Figure 4C). *P. berghei* infection caused significant decrease (*p* < 0.05) in hepatic GPx activity of negative control by over 211% compared to normal control on days 7 and 11 post-inoculation (Figure 4D), which was significantly reverted (*p* < 0.05) by over 200% to the range of normal control by doses of EA31 higher than 5 mg/kg body weight on days 7 and 11 post-inoculation (Figure 4D). *P. berghei* significantly reduced (*p* < 0.05) hepatic GR activity of negative control by 134.6% and 127.7% compared to normal control on days 7 and 11 post-inoculation respectively (Figure 4E). However, EA31 at doses higher than 10 mg/kg body weight significantly increased (*p* < 0.05) hepatic GR activity by over 70% compared to negative controls on days 7 and 11 post-inoculation (Figure 4E). Hepatic GSH concentration was significantly reduced (*p* < 0.05) by 57% after inoculation with *P. berghei* NK65 in negative control compared to normal control on day 7 post-inoculation while a similar trend was observed on day 11 post-inoculation. However, EA31 significantly increased (*p* < 0.05) hepatic GSH concentration by over 49.63% at doses higher than 10 mg/kg body weight compared to negative controls on days 7 and 11 post-inoculation (Figure 4F).

## Discussion

New ellagic acid hybrid compounds were envisioned to be synthesized and tested against *Plasmodium* species to improve their antimalarial properties. The synthesis of the molecule was monitored by TLC (Merck plates) stained with an acid solution of ceric sulphate and heated to 80°C. The product was purified by silica gel column chromatographic and fully characterized by NMR and IR spectrometric analysis. It was not a crystal but an amorphous solid, and we had no melting point. The NMR chemical shift for a carbonyl ester was observed at 174.75 (Supplementary Figure S3) and for the ellagic portion at 160.53 and 160.56 ppm (Supplementary Figure S3), confirming the ester bond formation and the ellagic acid portion (Supplementary Table S1) and at the IR spectra, the signals at 1,750–1,720 cm^–1^ (for C=O stretching) and at 1.300–1000cm^–1^ (for stretching vibration) confirm the ester bond formation, we saw the strong C-O stretching due to the two lactone groups from the ellagic acid portion (Supplementary Figure S4). The large signal at 3,500–3,000 cm^–1^ was be credited to the O-H groups with intra and intermolecular hydrogen bonding (Supplementary Figure S4). The peaks at 2,950–2,940 cm^–1^ were the stretching of the C-H bond with a bending band at 1,460–1,450 cm^–1^ and CH2 rock at 720 cm^–1^ (Supplementary Figure S4). The ester linker is susceptible to hydrolysis by acid, base, proteins (albumin) and enzymes (esterases) in physiological environment (Stefanidis and Jencks, 1993; Salvi et al., 1997; Tian and Stella, 2010; Fukami and Yokoi, 2012). Thus, the components in the hybrid compound will be hydrolyzed into the individual compounds *in vivo* to exert their respective pharmacological actions.

The results of this study revealed that EA31 was active against *P. falciparum* W2 (chloroquine-resistant clone), exhibiting the lowest IC_50_ (0.0072 μg/mL) compared to ellagic acid and artesunate. This showed that the hybrid compound possessed a higher antiplasmodial activity than its individual constituents. Hemozoin formation, the mechanism by which *Plasmodium* species detoxify free heme resulting from the digestion of hemoglobin in the food vacuole, is a confirmed target for many of the conventionally used antimalarials and adjudged to be an appropriate target for the development of new antimalarials (Kumar et al., 2007). Drugs, such as chloroquine and artesunate, have been reported to interfere with hemozoin formation (Pandey et al., 1999; Lin et al., 2015). In this study, EA31 was comparable to chloroquine in its β-hematin formation inhibitory activity, with an IC_50_ of 5.86 μg/mL. This suggests that the hybrid compound may effectively inhibit the biocrystalization of heme to hemozoin. The results also revealed that EA31 had the highest selectivity index compared to ellagic acid and artesunate, suggesting that it was less toxic than its parent compounds and is a good candidate for rational drug design.

For the suppressive studies, the significant dose-dependent increase in chemosuppression (>50%) on day 4 post-inoculation as well as the increase in mean survival time of EA31-treated group compared to artesunate- and ellagic acid-treated groups at doses higher than 5 mg/kg body weight, which compared favourably with chloroquine at higher doses, suggests that the hybrid compound was active against *P. berghei in vivo.* In absolute terms, EA31 was less active than artesunate but more active than ellagic acid at the same doses. However, the lower ED_50_ values for days 4 and 7 post-inoculation for suppressive test suggests that EA31 is a fast-acting compound, though it was higher than the reported oral ED_50_ value for chloroquine against *P. berghei* NK65 (1.9 mg/kg body weight; Deng et al., 1997) but lower than that of artesunate against drug sensitive *P. berghei* NY (12.66 mg/kg body weight; Vivas et al., 2007). Reports on the oral ED_50_ values for artesunate against *P. berghei* NK65 is scarce but comparing the results of this study with that of drug sensitive *P. berghei* NY, it was observed that EA31 was more potent than artesunate. Also, for the curative studies, EA31 caused significantly higher chemosuppression (>50%) compared to ellagic acid at all doses and artesunate at doses higher than 10 mg/kg body weight, comparing favourably well with chloroquine at the highest dose. This suggests that EA31 was active against established *P. berghei* infection in mice. Also, EA31 was less active than artesunate but more active than ellagic acid (which was partially active, causing 30.95% chemosuppression), at the same doses. Moreover, EA31 increased MST of mice with established infection compared to negative control and the parent compounds. Earlier reports have indicated that ellagic acid was less bioavailable (Borges et al., 2007). The observed increased antimalarial activity of EA31 compared to ellagic acid suggests that the synthesis of the two compounds into a single molecule alleviated this setback, thus potentiating the antimalarial activity of ellagic acid, though the antimalarial activity of artesunate was attenuated. As earlier mentioned, the hybrid compound has inhibition of hemozoin formation as one of its mechanisms of action; however, the possibility of having new targets cannot be ruled out. This might prevent the emergence of resistance of the parasite to the hybrid compound, thus favouring its use as a less toxic alternative. Varotti et al. (2008) also reported an enhanced antimalarial activity of MEFAS against established infection, a hybrid compound synthesized from artesunate and mefloquine, suggesting that synthesis of hybrid compounds from antimalarial compounds may enhance their activities. EA31was 6.5 times more active than amodiaquine-ellagic acid hybrid compound with an IC_50_ of 0.0072 μg/mL and 0.047 μg/mL respectively against *P. falciparum in vitro*. However, MEFAS was more active than EA31 with an IC_50_ of 0.001 μg/mL (Varotti et al., 2008; Żesławska et al., 2014).

Free radicals, especially reactive oxygen species (ROS), are normally generated in cellular systems. An imbalance between the free radical generating processes and the antioxidant system leads to oxidative stress, leading to chain reactions capable of damaging cells. Thus, antioxidants are crucial to the prevention of oxidative stress-mediated pathologies (Young and Woodside, 2001). Apart from physiological antioxidants, polyphenols have been found to play a prominent role in scavenging antioxidants. A good example of polyphenol is ellagic acid, which was used for the synthesis of the hybrid compound in this study. Ellagic acid is a known free radical scavenging polyphenol (Kilic et al., 2014) whereas artesunate is a prooxidant (Ackerman et al., 2009). The results of this study showed that EA31 had significantly lower DPPH and ABTS radical scavenging activities compared to ellagic acid and the standard, BHT. However, EA31 had higher NO scavenging activity and FRAP compared to BHT, comparing favourably with ellagic acid. It also exhibited higher total antioxidant capacity compared to quercetin, though lower than that of ellagic acid. The observed *in vitro* antioxidant activity of EA31 could be due to its ellagic acid component, which has been reported as a potent scavenger of DPPH, ABTS and superoxide radical (Kilic et al., 2014). It has also been reported that ellagic acid may hinder free radical production by Fe^3+^ *in vitro* by forming a complex with it (Dalvi et al., 2017). Thus, the antioxidant activity of EA31 may complement its antimalarial activity while treating malaria.

ROS generated during *Plasmodium* infection lead to increase in products of lipid peroxidation, such as MDA. Results obtained from this study revealed that MDA concentration was highly increased in the erythrocyte and liver of mice as a result of *P. berghei* NK65 infection, thus corroborating earlier reports (Scaccabarozzi et al., 2018). The elevated tissue MDA level recorded in negative control has been associated with increased metabolic rate of fast replicating parasites giving rise to large amount of ROS (Oluwatosin et al., 2014). The significant reduction in MDA concentration caused by the hybrid compound may not be as a result of the artesunate moiety because artesunate has been reported to form heme adducts (Haynes et al., 2014), which initiates a chain process leading to membrane lipid peroxidation and subsequently increased MDA level (Krishna et al., 2004). Farombi et al. (2015) also reported increased MDA level and H_2_O_2_ generation in the uterine and erythrocyte of mice treated with artemisinin. Majid et al. (1991) reported a reduction in ascorbate-dependent lipid peroxidation in microsomes isolated from the liver of mice after treatment with ellagic acid. Therefore, the reduction in lipid peroxidation product may be as a result of the ellagic acid constituent of EA31.

Superoxide dismutase (SOD) is primarily responsible for the detoxification of superoxide anion radical to hydrogen peroxide (H_2_O_2_) and is the firstline enzymic antioxidant defense in cellular systems. H_2_O_2_ is toxic and is, in turn, detoxified to H_2_O by catalase and glutathione peroxidase, thus preventing the toxic effects of superoxide anion in the cell (Hussein et al., 2016). The decrease in SOD activity in the negative control may be due to the parasite triggering the release of large amount of superoxide anion radicals which has overwhelmed the buffering mechanism of the antioxidant defense of the host (Okpoghono and Osioma, 2012). Thus, the increase in SOD activity caused by the hybrid compound may be due to increased synthesis of the enzyme or activation of the enzyme *in situ* (Balogun et al., 2014). Moreover, the ellagic acid component of EA31 may also spare the utilization of SOD through its antioxidant activity, thereby preventing the depletion of the enzyme.

Catalase (CAT) is a tetrameric hemoprotein abundant in tissues and erythrocytes. In the presence of H_2_O_2_, CAT undergoes divalent redox reaction at its active site. It functions at high concentration of H_2_O_2_ (Olatunde Farombi et al., 2003). Decrease in erythrocyte CAT activity observed in negative control may result from inactivation of the enzyme by high level of ROS (Adewole and Adebayo, 2017) or utilization of erythrocytic proteins by the parasite (Balogun et al., 2014). Also, the reduced liver CAT activity observed in negative control may result from overstressing the buffering mechanism of the antioxidant defense of the host (Okpoghono and Osioma, 2012). The increase in activity of this enzyme after treatment with EA31 compared to negative control may be due to induction of its synthesis, its activation *in situ* or the enzyme being spared by the antioxidant activity of ellagic acid present in the hybrid compound (Mohan et al., 1992).

Glutathione peroxidase (GPx) catalyzes the detoxification of hydrogen peroxides and lipid hydroperoxides to water (Ahmad et al., 2010). Catalase and GPx can both catalyze the conversion of hydrogen peroxide to water and oxygen. However, GPx preferentially acts at low concentration of H_2_O_2_ (Casado et al., 1995). *P. berghei* NK65 infection-induced reduction in GPx activities in the erythrocyte and liver of negative control corroborates findings of previous studies (Adebayo et al., 2017). The reduction in GPx activity suggests impaired utilization of GSH in parasitized erythrocytes and in the liver. The increase in liver glutathione peroxidase activity in the liver of EA31-treated mice compared to negative control may result from a compensatory mechanism to synthesize more of the enzyme in order to offset the oxidative stress (Balogun et al., 2014). It could also be as a result of the enzyme in the erythrocyte and liver being spared by the antioxidant activity of the ellagic acid component of the hybrid compound or being activated *in situ* (Balogun et al., 2014).

Glutathione reductase (GR) is an enzyme that helps in the maintenance of sufficient amount of reduced glutathione in cells. GR is a homodimer which uses NADPH to reduce glutathione disulfide to GSH (Sarma et al., 2003). The significant reduction observed in erythrocyte and liver GR activities in the negative control could be as a result of the overwhelming of its buffering mechanism in maintaining glutathione in its reduced state (GSH) due to the parasite-imposed oxidative stress. The increased erythrocyte and liver GR activity observed in EA31-treated mice compared to negative control may be due to activation of the enzyme *in situ* or induction of its synthesis for the transformation of GSSG to GSH, in order to combat the increased generation of ROS by the parasite.

The mechanism by which ellagic acid induced the antioxidant defense system could be by activating the Nuclear factor erythroid 2-related factor 2 (Nrf2)/antioxidant response elements (ARE) signaling pathway because it has been reported that ellegic acid activates this signaling pathway (Zhang et al., 2022). When there is oxidative stress, Nrf2 dissociates from Kelch-like ECH-associated protein 1 (Kaspar et al., 2009; Milković et al., 2019). It enters the nucleus and combines with ARE and induces the synthesis of SOD and CAT (Lu et al., 2016). The downstream glutathione peroxidase is also activated.

Reduced glutathione (GSH) is a nucleophile and powerful antioxidant which is crucial for protective processes in cellular systems such as xenobiotic conjugation and excretion, inflammatory cytokine cascade control and ROS detoxification (Brown et al., 2004). Increased utilization of GSH resulting from excessive ROS production has been earlier reported to be responsible for the observed decrease in tissue GSH level in infected mice (Halliwell, 2007). The observed increase in reduced glutathione (GSH) upon treatment with EA31 may not be as a result of its artesunate component because artemisinin derivatives have been reported to deplete intracellular levels of glutathione (Smith et al., 2001). It could however be as a result of the ellagic acid component because it has been shown that ellagic acid increased GSH level in the liver of mice (Majid et al., 1991).

## Conclusion

The results revealed that EA31 had higher activity than artesunate against *P. falciparum* W2 and favourably compared with it *in vivo*. The hybrid formation did not interfere with the activity of the artesunate component of the hybrid compound in inhibiting hemozoin formation. The hybrid compound possessed another advantage of inducing the antioxidant defense system in the erythrocyte and liver against malaria-induced oxidative stress. The results of this study suggest that artesunate-ellagic acid hybrid compound at 10 mg/kg body weight may be a better treatment option for malaria compared to artesunate (4 mg/kg body weight recommended daily dose), possessing the ability to directly kill the parasite and reduce the accumulation of reactive oxygen species which is responsible for the secondary complications of malaria.

## Data Availability

The original contributions presented in the study are included in the article/Supplementary Material, further inquiries can be directed to the corresponding author.
