# Supplementary figures and images for: Antimalarial and antioxidant activities of novel artesunate-ellagic acid hybrid compound in vitro and in vivo
Source: Front Pharmacol. 2024 Jun 18;15:1192659. doi: 10.3389/fphar.2024.1192659 (PMC11217523; doi:10.3389/fphar.2024.1192659)

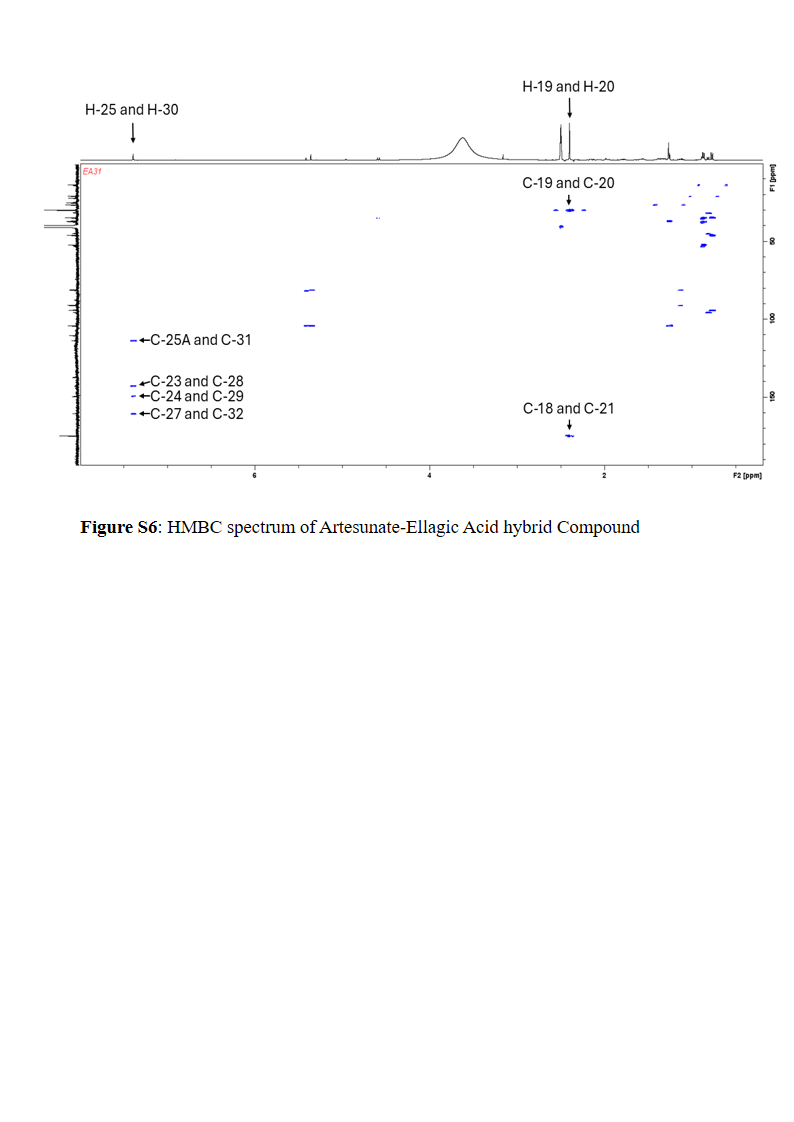

Supplement: Supplementary file 1 [file Image6.TIF]

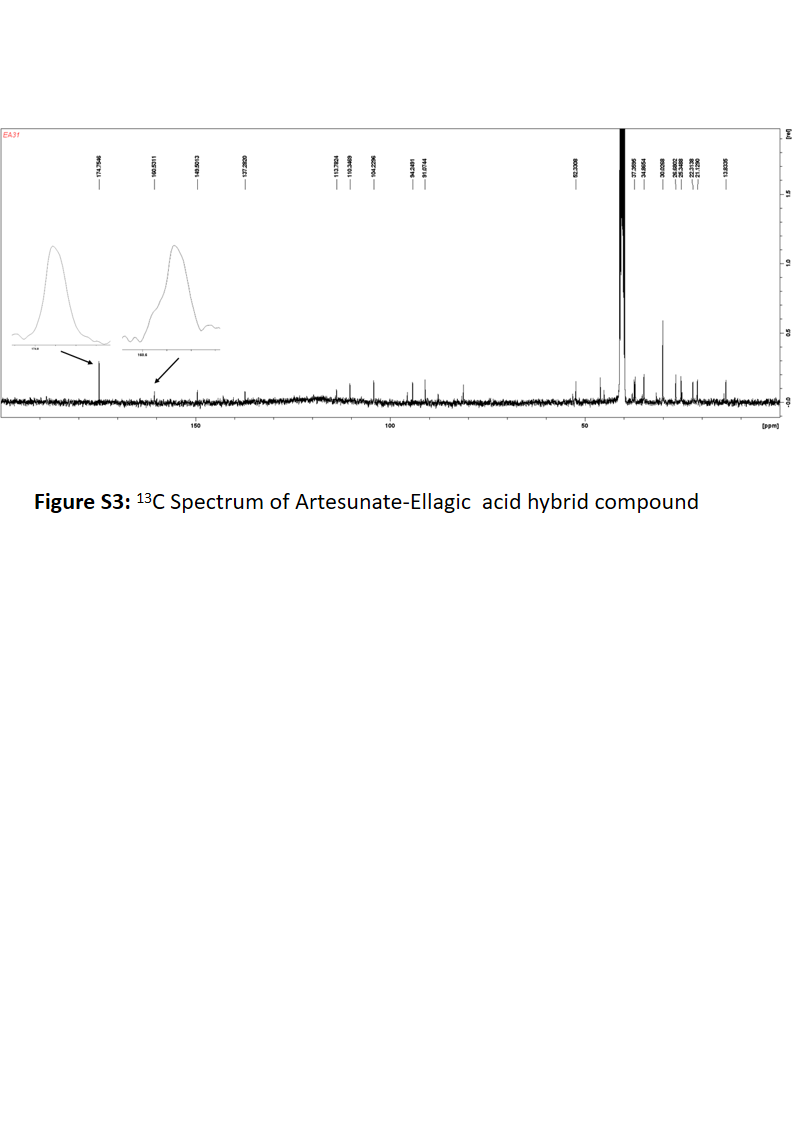

Supplement: Supplementary file 3 [file Image3.TIF]

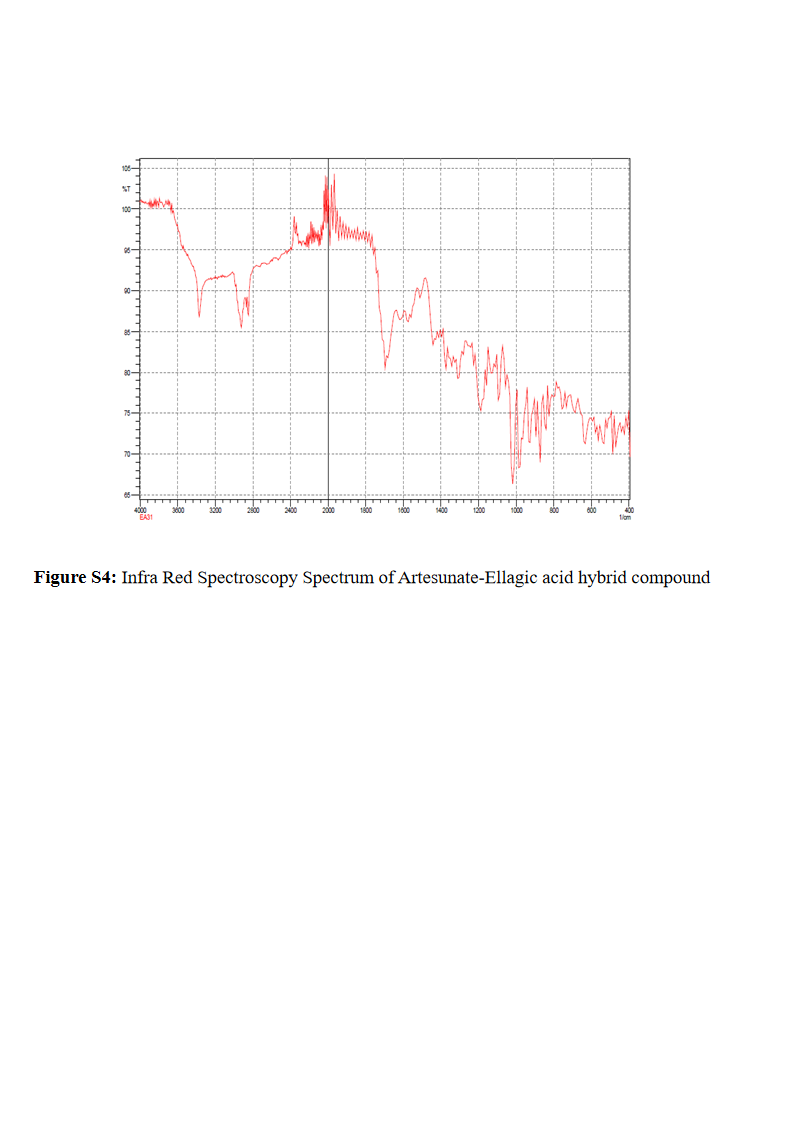

Supplement: Supplementary file 4 [file Image4.TIF]

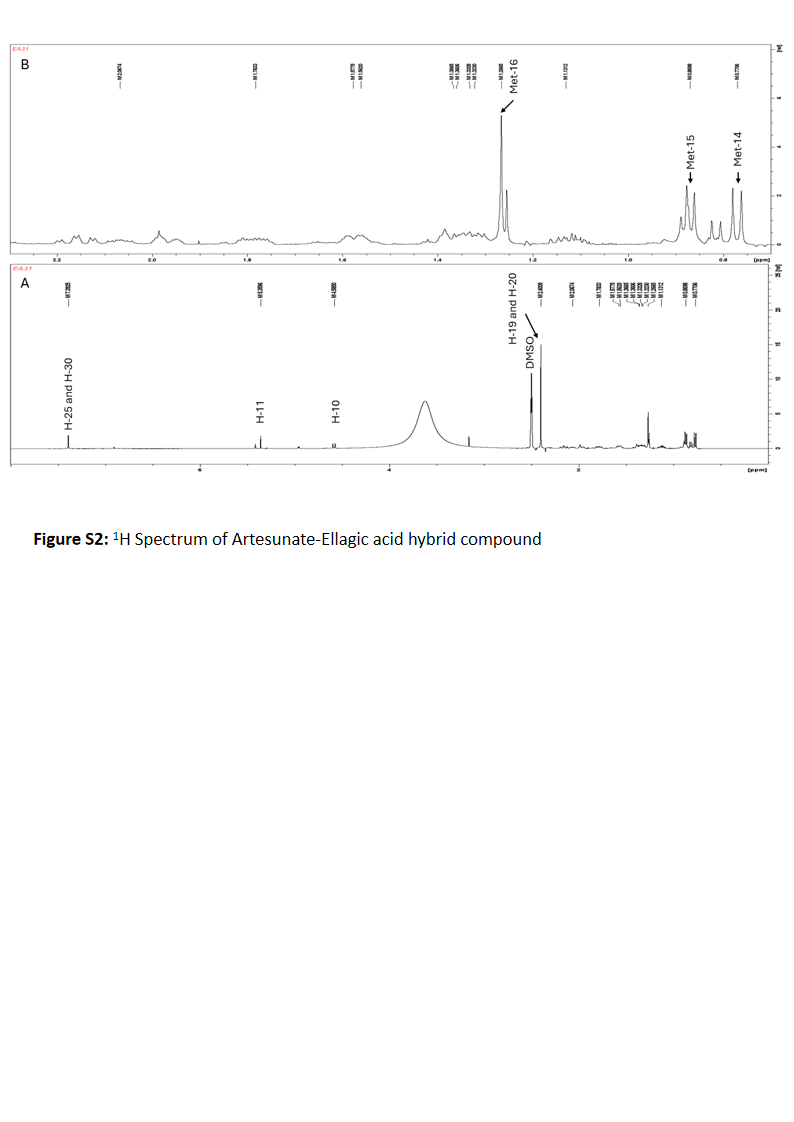

Supplement: Supplementary file 5 [file Image2.TIF]

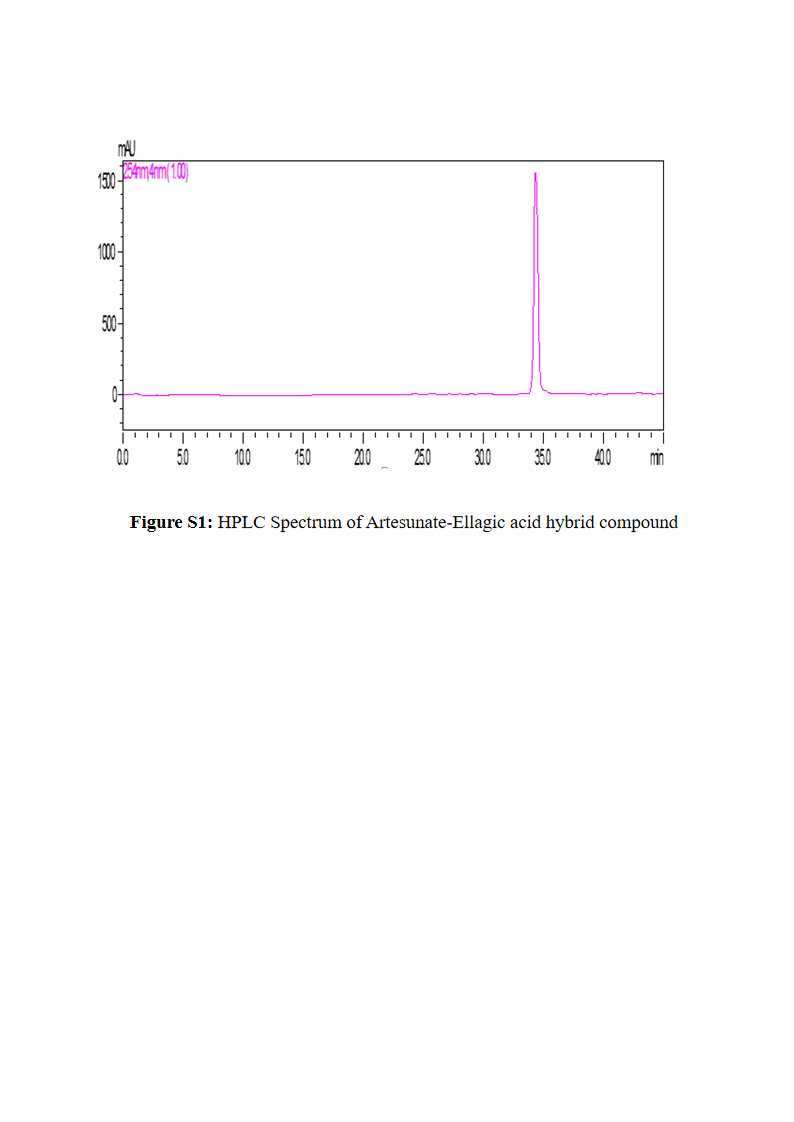

Supplement: Supplementary file 6 [file Image1.TIF]

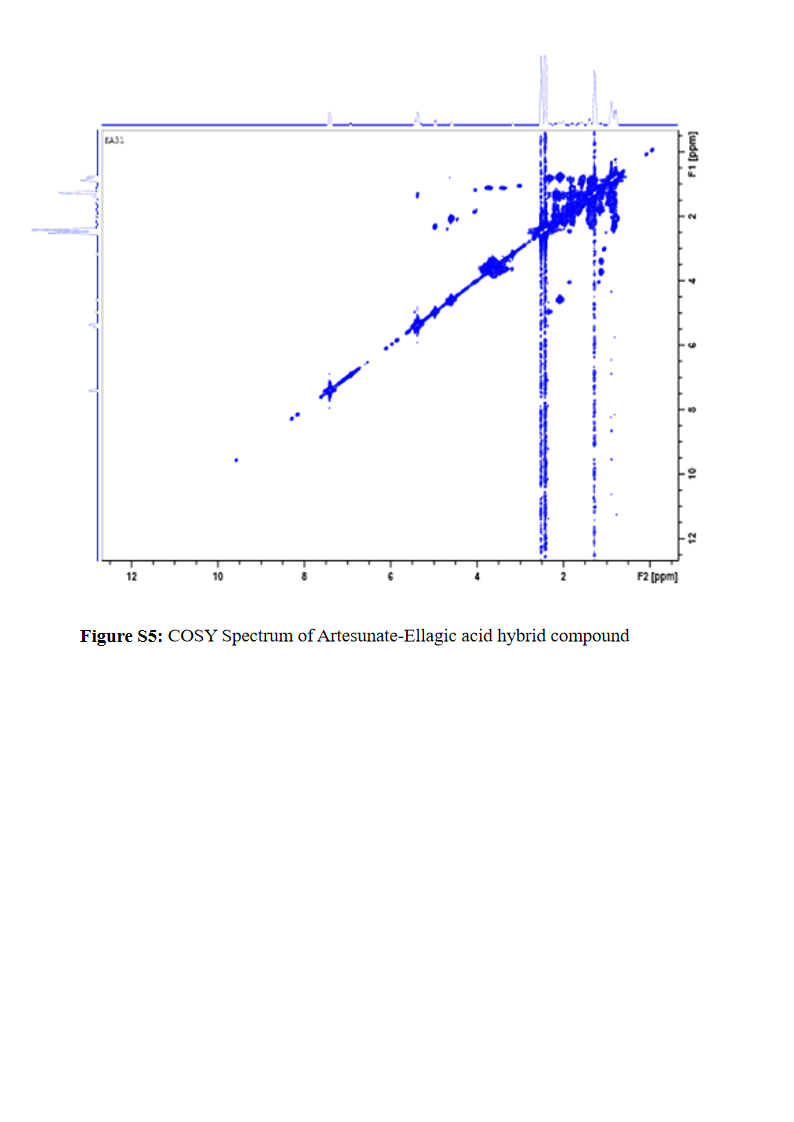

Supplement: Supplementary file 7 [file Image5.TIF]
