# Supplementary material for: Antimalarial and antioxidant activities of novel artesunate-ellagic acid hybrid compound in vitro and in vivo
Source: Front Pharmacol. 2024 Jun 18;15:1192659. doi: 10.3389/fphar.2024.1192659 (PMC11217523; doi:10.3389/fphar.2024.1192659)
Supplement: Supplementary file 2 [file Table1.docx]

Supplementary Material

Table S1: NMR data (400MHz) of EA31 in DMSO d_6_ (^1^H-^13^C HSQC, ^1^H-^1^H COSY and ^1^H-^13^C HMBC)

| **Position** | **HSQC** | | **COSY** | **HMBC** | | |
| --- | --- | --- | --- | --- | --- | --- |
|  | ***δ* _C_** | ***δ* _H_** | **^2-4^*J*_H,H_** | **^2^*J*_C,H_** | **^3^*J*_C,H_** | **^4^*J*_C,H_** |
| **3** | 104.22 | - | - | Me-16; H-4 | H-5; H-11 | H-5a |
| **4** | 25.34 | 1.78 (m)  1,33 (m) | H-4; H-5; H-5a; Me-16 | H-5 | H-5a; Me-16 | H-6 |
| **5** | 22.31 | 1,57 (m)  1.36 (m) | H-4; H-5; H-5a | H-4; H-5a | H-6 | Me-15; Me-16 |
| **5a** | 52.33 | 1.13 (m) | H-4; H-5; H-6; H-7; H-11; H-8a; Me-15 | H-5; H-6 | H-4; H-7; H-8a; H-11; Me-15 | H-8; H-9 |
| **6** | 34.92 | 1.56 (m) | H-5; H-5a; H-7; H-8; Me-15 | H-5a; H-7; Me-15 | H-5; H-8 | H-4; H-8a; H-11 |
| **7** | 22.31 | 1.55 (m)  1.32 (m) | H-5a; H-6; H-7; H-8; H-8a; Me-15 | H-6; H-8 | H-5a; H-8a; Me-15 | H-5; H-9 |
| **8** | 22.31 | 1.55 (m)  1.32 (m) | H-6; H-7; H-8; H-8a; H-9 | H-7; H-8a | H-6; H-9 | H-5a; H-10; H-11; Me-14; Me-15 |
| **8a** | 37.36 | 1.32 (m) | H-5a; H-7; H-8; H-8a; H-9; H-10; H-11; Me-14 | H-8; H-9 | H-5a; H-7; H-10; H-11; Me-14 | H-5; H-6 |
| **9** | 34.86 | 2.06 (m) | H-8; H-8a; H-10; Me-14 | H-8a; H-10; Me-14 | H-7 | H-5a; H-7; H-11 |
| **10** | 94.24 | 4.58 (d, *J* 9.00 Hz) | H-8a; H-9; H-11; Me-14 | H-9 | H-8a; H-11; Me-14 | H-8; H-19 |
| **11** | 91.07 | 5.36 (s) | H-5a; H-8a; H-10 | - | H-5a; H-8a; H-10; | H-4; H-5; H-6; H-8; H-9; Me-16 |
| **11a** | 90.13 | - | - | H-5a; H-8a; H-11 | H-5; H-6; H-7; H-8; H-9 | H-4; H-7; H-11; Me-14; Me-15 |
| **18; 21** | 174.75 | - | - | H-19; H-20 | H-19; H-20 | H-9 |
| **19; 20** | 30.02 | 2.40 (s) | H-20 | H-19; H-20 | - | H-10 |
| **23; 28** | 142.92 | - | - | - | H-25; H-30 | - |
| **24; 29** | 149.50 | - | - | H-25; H-30 | - | - |
| **25; 30** | 110.34 | 7.39 (s) | - | - | - | - |
| **25a; 31** | 113.78 | - | - | H-25; H-30 | - | - |
| **26; 31a** | 104.14 | - | - | - | H-25 | H-30 |
| **27; 32** | 160.53 | - | - | - | H-25; H-30 | - |
| **28a; 32a** | 137.28 | - | - | - | - | H-25; H-30 |
| **Me-14** | 13.83 | 0.77 (d, *J* 7.20 Hz) | H-9 | H-9 | - | - |
| **Me-15** | 21.14 | 0.86 (d, *J* 6.34 Hz) | H-6 | H-6 | - | - |
| **Me-16** | 26.68 | 1.26 (s) | - | - | - | - |

Table S2: Parasitemia in *P. berghei* NK-65 infected mice treated with artesunate- ellagic acid combination (Ratio 1:1, EA01)

|  | Parasitemia (% chemosuppression) | | |  |
| --- | --- | --- | --- | --- |
| Groups | Day 4* | Day 6* | Day 8* | MST |
| Control  (Untreated) | 0.97 | 4.03 | 5.56 | 14 |
| 20 mg/kg b.w.  Chloroquine | 0.06 (93.81) | 0.45 (88.83) | 0.52 (90.65) | 22 |
| 4 mg/kg b.w.  Artesunate | 0.27 (72.16) | 0.89 (77.92) | 1.92 (65.47) | 19 |
| 10 mg/kg b.w.  Ellagic Acid | 0.35 (63.92) | 2.11 (47.64) | 3.34 (39.93) | 21 |
| 5 mg/kg b.w.  EA01 | 0.45 (53.61) | 1.95 (51.61) | 3.30 (40.65) | 25 |
| 10 mg/kg b.w.  EA01 | 0.33 (65.98) | 1.73 (57.07) | 2.54 (54.32) | 26 |
| 20 mg/kg b.w.  EA01 | 0.30 (69.07) | 1.62 (59.88) | 1.97 (64.57) | 25 |
| 40 mg/kg b.w.  EA01 | 0.26 (73.20) | 1.38 (65.76) | 1.82 (67.27) | 28 |
| 80 mg/kg b.w.  EA01 | 0.21 (78.35) | 0.92 (77.17) | 1.68 (69.18) | 26 |

Values are means of 5 replicates. *Day post-inoculation; b.w.: body weight

Table S3: Parasitemia in *P. berghei* NK-65 infected mice treated with artesunate- ellagic acid combination (Ratio 2:1, EA02)

|  | Parasitemia (% chemosuppression) | | |  |
| --- | --- | --- | --- | --- |
| Groups | Day 4* | Day 6* | Day 8* | MST |
| Control  (Untreated) | 0.97 | 4.03 | 5.56 | 14 |
| 20 mg/kg b.w.  Chloroquine | 0.06 (93.81) | 0.45 (88.83) | 0.52 (90.65) | 22 |
| 4 mg/kg b.w.  Artesunate | 0.27 (72.16) | 0.89 (77.92) | 1.92 (65.47) | 19 |
| 10 mg/kg b.w.  Ellagic Acid | 0.35 (63.92) | 2.11 (47.64) | 3.34 (39.93) | 21 |
| 5 mg/kg b.w.  EA02 | 0.55 (43.30) | 2.01 (50.12) | 3.01 (45.86) | 24 |
| 10 mg/kg b.w.  EA02 | 0.47 (51.54) | 1.93 (52.10) | 2.85 (48.74) | 27 |
| 20 mg/kg b.w.  EA02 | 0.38 (60.82) | 1.35 (66.50) | 2.78 (50.00) | 28 |
| 40 mg/kg b.w.  EA02 | 0.41 (57.73) | 1.21 (70.00) | 2.41 (56.56) | 28 |
| 80 mg/kg b.w.  EA02 | 0.32 (67.01) | 1.60 (60.30) | 2.36 (57.55) | 20 |

Values are means of 5 replicates. *Day post-inoculation; b.w.: body weight

Table S4: Parasitemia in *P. berghei* NK-65 infected mice treated with artesunate-ellagic acid combination (Ratio 1:2, EA03)

|  | Parasitemia % (% chemosuppression) | | |  |
| --- | --- | --- | --- | --- |
| Groups | Day 4* | Day 6* | Day 8* | MST |
| Control (Untreated) | 0.97 | 4.03 | 5.56 | 14 |
| 20 mg/kg b.w.  Chloroquine | 0.06 (93.81) | 0.45 (88.83) | 0.52 (90.65) | 22 |
| 4 mg/kg b.w.  Artesunate | 0.27 (72.16) | 0.89 (77.92) | 1.92 (65.47) | 19 |
| 10 mg/kg b.w.  Ellagic Acid | 0.35 (63.92) | 2.11 (47.64) | 3.34 (39.93) | 21 |
| 5 mg/kg b.w.  EA03 | 0.52 (46.39) | 2.14 (46.89) | 3.24 (41.73) | 25 |
| 10 mg/kg b.w.  EA03 | 0.41 (58.76) | 1.71 (57.57) | 2.38 (57.19) | 20 |
| 20 mg/kg b.w.  EA03 | 0.37 (61.86) | 1.38 (65.76) | 2.02 (63.67) | 25 |
| 40 mg/kg b.w.  EA03 | 0.29 (70.10) | 1.15 (71.46) | 1.75 (68.53) | 23 |
| 80 mg/kg b.w.  EA03 | 0.34 (64.95) | 1.60 (60.30) | 1.52 (72.66) | 19 |

Values are means of 5 replicates. *Day post-inoculation; b.w.: body weight
